# Corticobasal manifestations of Creutzfeldt-Jakob disease with D178N-homozygous 129M genotype

**DOI:** 10.1080/19336896.2020.1812367

**Published:** 2020-09-18

**Authors:** Yumeng Huang, Ma Jianfang, Rodrigo Morales, Huidong Tang

**Affiliations:** a Department of Neurology, Shanghai Jiao Tong University Medical School Affiliated Ruijin Hospital, Shanghai, China; b Department of Neurology, McGovern Medical School, the University of Texas Health Science Center at Houston, Houston, TX, USA; c Centro Integrativo de Biologia y Quimica Aplicada (CIBQA). Universidad Bernardo OHiggins, Santiago, Chile

**Keywords:** Creutzfeldt-Jakob disease, D178N-129M, fatal familial insomnia

## Abstract

Creutzfeldt-Jakob disease (CJD) is a prion disease, usually presented with memory loss, ataxia, dementia, myoclonus, involuntary movements and psychiatric problems. D178N-homozygous 129M genotype has been recognized in the diagnosis of fatal familial insomnia (FFI) globally. Here we report a patient presented with progressive left upper limb stiffness, bradykinesia, hypomimia and weight loss (10 kg) initially. She progressed to dementia, dysphasia, dysphonia and be bedridden quickly but did not present insomnia. She was diagnosed with CJD corticobasal subtype carrying a classic D178N-129M mutation of *PRNP* in FFI. Remarkably, she has a strong family history of neurological degeneration diseases but the other members of this pedigree who do not carry D178N-homozygous 129M mutation in *PRNP* do not present any CJD or FFI symptoms. We conclude that this patient carrying D178N-homozygous 129M mutation in *PRNP* should be diagnosed as CJD. Thus, the clinicopathology should be considered as a crucial evidence in diagnosing some cases, but FFI could be evaluated as a differential diagnosis with a unique clinical profile.

**List of abbreviations**

AD: Alzheimer disease; ADL: Activities of Daily Living; CBD Cortical basal degeneration; CBS: Corticobasal syndrome; CJD: Creutzfeldt-Jakob disease; DWI: Diffusion-weighted image; EEG: Electroencephalograph, fCJD: familial Creutzfeld-Jakob disease; FFI: Fatal familial insomnia; FLAIR: Fluid-attenuated inversion recovery; MMSE: Mini-mental state examination; MoCA: Montreal Cognitive Assessment; MRI: Magnetic resonance imaging; PD: Parkinson disease; PrP: Prion protein; PSWC: Periodic sharp wave complexes; SWI: Susceptibility-weighted imaging

## Background

Creutzfeldt-Jakob disease (CJD) is a prion disease, usually presented with memory loss, ataxia, dementia, myoclonus, involuntary movements and psychiatric problems [1]. Presently, clinical history, MRI (Magnetic Resonance Imaging) and/or 14-3-3 protein content in cerebrospinal fluid are recommended for CJD diagnosis [2,3]. Over 60 *PRNP* gene mutations have been reported for genetic CJD, including missense, deletion, insertion and amber mutations [1], in which E219K and E200K are mostly highlighted [4]. D178N, a missense mutation on codon 178 of *PRNP* (D178N) with the substitute of asparagine for aspartic acid, has been associated with the clinicopathological phenotype of either CJD or fatal familial insomnia (FFI) depending on the polymorphic change of the prion protein (PrP) at position 129 (D178N-129M/M is related to FFI while D178N-129 V/V related to CJD) [5–9]. Here, we report a patient with D178N-129M/M genotype clinically manifesting corticobasal manifestations of CJD. Moreover, she lives more than 12 months which is remarkable in familial CJD (fCJD). This finding is unusual, we deduct that it might be due to a much higher base of 129M polymorphism in East Asian population than Caucasian, which makes the clinical presentation of D178N-129M/M genotype more complicated.

## Clinical presentation

A 58-year-old female presented with progressive left upper limb stiffness, bradykinesia, hypomimia and weight loss (10 kg) in a period of 6 months. She denied insomnia. Physical examination revealed her wrist overextended with swelling and pain, muscle strength was 4/5 and muscle tension was increased in the left arm. The deep tendon reflex was 2+ of bilateral lower limbs, and left ankle clonus was positive. Palm jaw test was positive bilaterally. Other neurological examination was unremarkable.

Cognitive functions were evaluated by different scales in October 2018. These included mini-mental state examination (MMSE) (19 point: orientation 8/10, attention and calculation 0/5, memory and recall 4/6, language 7/9), Montreal Cognitive Assessment (MoCA) (14 points: orientation 5/6, executive function/visuospatial ability 0/5, clock-drawing test 0/3, animal naming 3/3, memory 1/2, attention 2/7, language abilities 1/3, abstraction 1/2), and Hamilton depression questionnaire 5/68 points and Activities of Daily Living (ADL) 54/77 points.

In the next 12 months’ follow-up, she developed bilateral arm spasm, dysphonia and bedridden. She also presented deterioration of dysphasia, appetite loss and required nasal fed. However, the swollen wrist was relieved spontaneously several months prior follow-up examination. Her ADL declined at a fast rate, scoring 1/77 in November 2019. Since the patient manifested dysphonia and was fixed in bed, we were not able to evaluate other cognitive and mood scales.

The complete blood count, serum biochemistry and hepatitis virus and whole immunology tests were in normal ranges except for a moderate increased serum rheumatoid factor (45 IU/ml, normal limits: 0–20 IU/ml). This can help to exclude autoimmune disease and infections.

**Figure 1.**
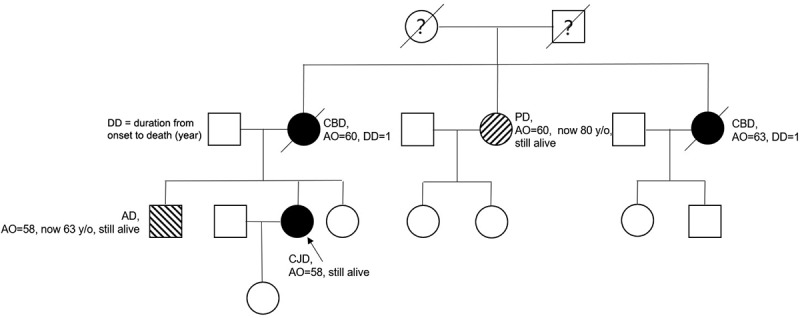
Pedigree of a Chinese genetic CJD case.

The patient had a positive family history for neurodegenerative diseases: her mother presented with similar symptoms at the age of 60 and was diagnosed as possible CBD (cortical basal degeneration), dying 1 year later without genetic tests being performed. One of her aunts had the same problem with the onset of clinical symptoms manifested at 50-years old, surviving 1 year after. Another aunt was diagnosed with Parkinson disease (PD) at the age of 60 and still alive at the moment of writing this article (80-years old). Her brother presented with memory loss at age 58 and was later diagnosed with Alzheimer disease (AD) (Figure 1. Pedigree of patient’s family).

AO = age of onset; DO = duration from onset to death (year). Circles indicate females; squares, males; shaded symbols, affected individuals; stripes within a symbol, related neuropathy; diagonal bar, deceased members; black arrow, the pro-band. The maternal mother (II:1) presented with similar symptoms as the pro-band at the age of 60 and was diagnosed as possible CBD (cortical basal degeneration) who died 1 year after onset without genetic tests being performed. Subject II:3 had the same problem at age 50 and died 1 year later. Subject II:2, diagnosed with Parkinson’s disease at age 60, was alive at the moment of writing (80-years old). Subject III:1 presented with memory loss initially and was diagnosed as Alzheimer’s disease. The latter patient displayed a genetic result of p.178 GM/M in *PRNP* (wild type). Subject III:3 was healthy but refused to do a gene test. Subject IV:1 did not show any neurotic symptoms and her gene report was p.178 GM/M in PRNP (wild type).

## Investigation

**Figure 2.**
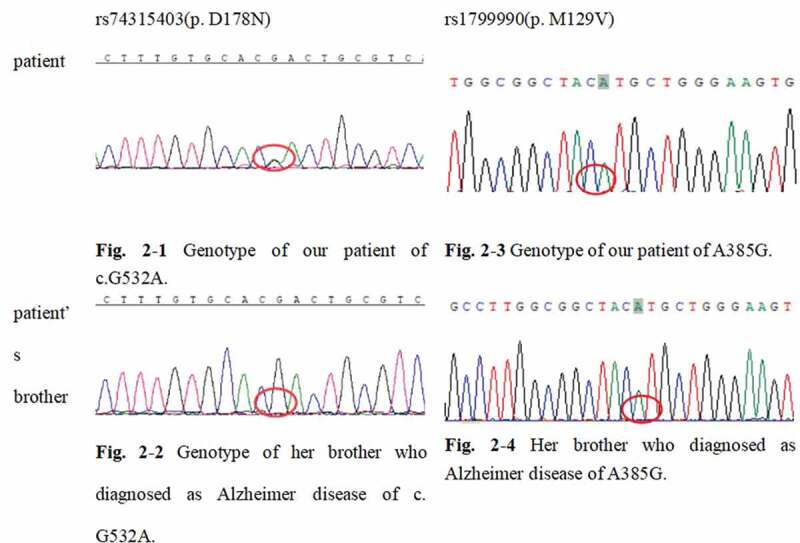
Gene report of the patient and her brother.

For genetic sequencing, genomic DNA was extracted from peripheral blood. Firstly, whole-exome sequencing of our patient was tested (RayLee Biotech co., Shanghai). Next, her brother’s peripheral blood sample was confirmed for the mutation and polymorphism spot. The standard *PRNP* sequence (NCBI: NM_000311) was compared to detect whether there was a mutation in the *PRNP* gene and the polymorphism of the 129 codon. Our patient’s gene report unveiled a c.G532A/p.D178N mutation in the *PRNP* gene. The patient was also *PRNP* A385G./p.129M (Figure 2). Her brother (who had diagnosed with Alzheimer’s disease at 58-years old and was 63-years old at the time of manuscript writing) and daughter (healthy) were wild types for the *PRNP* gene (p.178 N and A385G./p.129M).

(a) Genotype of our patient of c.G532A. (b) Genotype of her brother who diagnosed as Alzheimer disease of c. G532A. (c) Genotype of our patient of A385G. (d) Her brother who diagnosed as Alzheimer disease of A385G.

**Figure 3.**
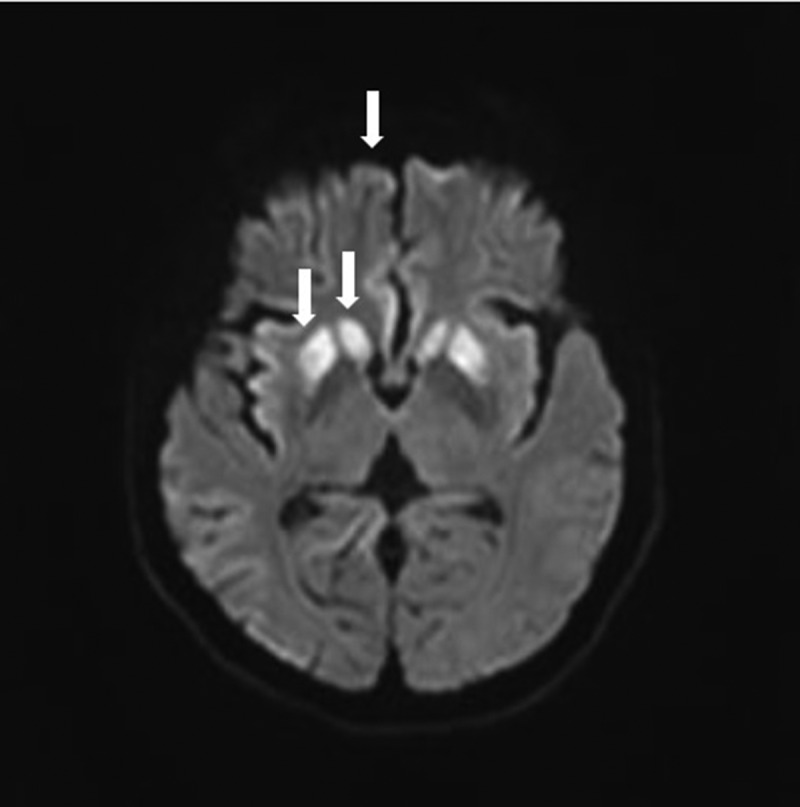
MRI with DWI image of the patient.

**Figure 4.**
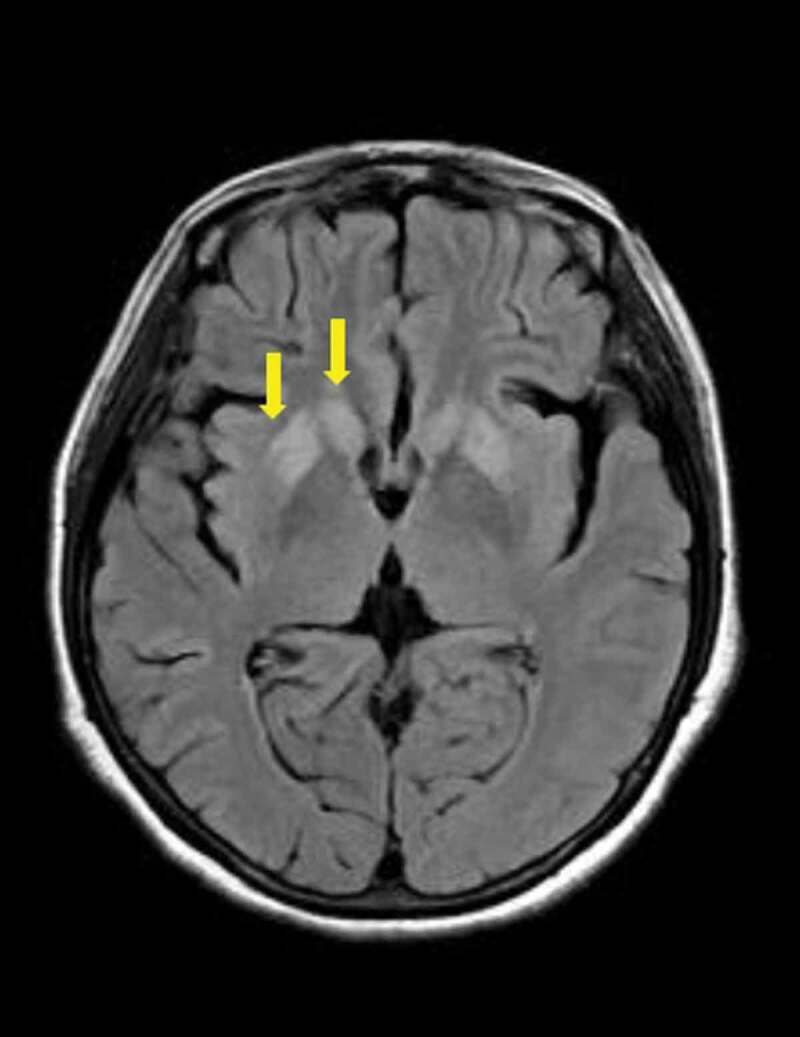
MRI with T2-FLAIR image of the patient.

Magnetic resonance imaging (MRI) plus susceptibility-weighted imaging (SWI) with vascular remoulding of brain (during the initial admission) showed no microbleeds. Diffusion-weighted imaging (DWI) sequence showed symmetrically hyperintensity in basal ganglion (especially putamen and the head of caudate) and medial frontal lobe cortices (Figure 3). T2-FLAIR images suggest the involvement of the putamen and caudate (Figure 4).

MRI with DWI shows obvious hyperintensity in the bilateral caudate and putamen and slight hyperintensity in the medial frontal lobe cortices (the right side hyperintensed putamen, caudate and frontal lobe cortices are pointed with white arrows).

T2-FLAIR images with characteristic involvement of the putamen and caudate (Figure 3, the right side involved putamen and caudate are pointed with yellow arrows).

**Figure 5.**
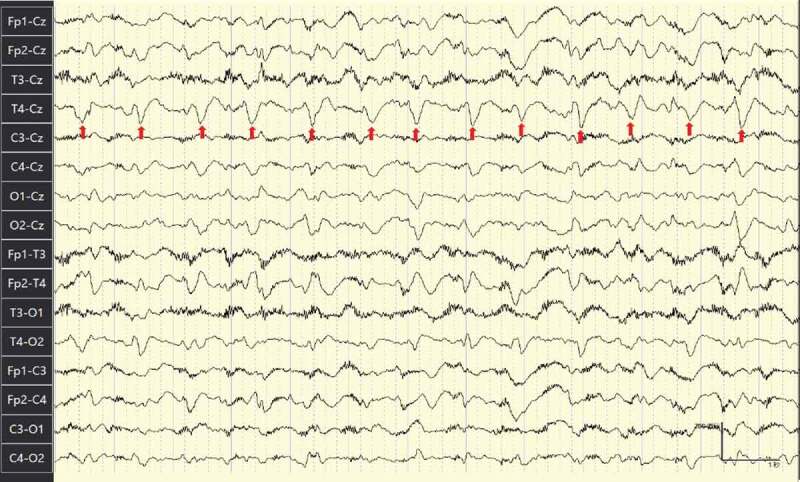
EEG of the patient.

EEG (Electroencephalograph) (Figure 5. EEG at 2 months after the initial admission) showed periodic synchronous diffuse slow wave and frequent sharp wave and periodic sharp wave complexes (PSWC) pattern.

EEG taken after 2 months of initial admission reported diffuse slow wave and frequent sharp wave and sharp slow wave complex pattern (with red arrows), more prominent in the right hemisphere.

Polysomnography was also attempted. Unfortunately, the patient was not able to cooperate. In that sense, this parameter was not evaluated.

## Diagnosis and outcomes

She was diagnosed corticobasal degeneration during initial admission and treated with Baclofen 10 mg Bid and Madopar with a maximum dose of 250 mg TID to relieve her symptoms but without any improvement. After we got her gene report, we diagnosed her with genetic CJD. She tapered Baclofen and Madopar after being discharged from the hospital. In the next 12 months’ follow-up, her progression was rapid. She got bedridden with other previous symptoms deteriorated (as described in the clinical presentation part). However, the swollen wrist was relieved spontaneously. Even though the patient family has hope in special therapy but due to the limited options for fCJD we could provide little treatment for her. We provided suggestions for her daily care, and gene tests and consults for her family.

## Discussion and conclusion

Here, we report a typical CJD patient who presented with corticobasal manifestations. Genetic report of *PRNP* displayed the disease-associated D178N-129M mutation. Based on previous studies, it is acknowledged that *PRNP* D178N-129M haplotype is prone to manifest as FFI, while the D178N-129V trait is linked to CJD [5,6,10,11]. Nevertheless, the definite pattern of D178N-129M genotype-phenotype is not assured. As the patient described in this report displayed the D178N-129M genotype and should be suspicious of FFI, she did not develop any problems with her sleeping behaviour, while still had a typical clinical presentation of corticobasal syndrome which is very reasonable for fCJD diagnoses. Previous reports described several clinical cases of D178N patients in with clinicopathological manifestation of CJD [4,10,12–15]. Table 1 summarizes the cases described to date which contains patients carrying D178N-129M of *PRNP* gene and displaying different prion disease phenotypes.

**Table 1.** Cases reported patients carrying D178N-129M of *PRNP* gene and displaying a variety of prion disease phenotypes.

Study	Origin	Genotype	Clinical profile
Medori et al.,1992^[19]^	An American family	a kindred patients with D178N(129 codon not clear)	FFI
Medori et al.,1993^[20]^	A French family	3 patients of a kindred with D178N-129M/M, 2 patients of the kindred with D178N-129M/V	All patients presented with FFI
Reder et al.,1995^[21]^	American	1 patient with D178N-129M/M	FFI
McLean et al.,1997^[6]^	An Austrilian family	6 patients with D178N-129M/M from one kindred	1 presented with CJD (D178N-129M/M) phenotype and 4 with FFI phenotype(D178N-129M/M)
Zerr et al.,1998^[16]^	German	8 patients with D178N-129M	The clinical course of all these patients resembled sporadic CJD. Within 6 acquired brain autopsy, 1 neuropathologic examination showed changes that were more reminiscent of forms of sporadic CJD; the remaining 5, the histopathology was typical of FFI.
Harder et al.,1999^[9]^	German	7 patients with D178N, including 5 patients with 129M/M,2 patients with 129M/V	7 genetic diagnosis of FFI, but clinical diagnosis with CJD, FFI, AD, GSS,etc
Taniwaki et al.,2000^[10]^	A Japanese family	3 patients with D178N-129M	3 patients with cerebral ataxia without overt insomnia diagnosed fCJD
Dauvilliers et al.,2004^[22]^	French	1 patient with D178N-129M/M	FFI presented with circadian rhythms changes
Spacey et al.,2004^[23]^	A family of Chinese descent	1 patient with D178N-129M/M, 1 patietn genotype unclear	2 patients from this kindred were FFI
Zarranz et al.,2005^[12]^	Spanish (Basque born families)	17 patients carrying D178N-129M	7 out of 17 patients has CJD phenotype
Synofzik et al.,2009^[17]^	A German family	all with D178N but 129 codon was not all clear demonstrated	1GSS with D178N-M129V, 2 CJD, 1 FFI, 1 atypical Alzheimer, 1 Freidreich ataxia, 1 brain degeneration, 1 brain softening, 1 asymptomatic member with D178N-129M
Saitoh et al.,2010^[24]^	Japanese	2 patients with D178N-129M/M	1 CJD(D178N-129M/M) phenotype and 1 FFI phenotype(D178N-129M/M) with the same PrP^sc^ ratio glycoform
Lin et al.,2015^[7]^	Chinese	1 patient with D178N-129M	1 CJD phenotype
Megelin et al.,2017^[25]^	A French family	3 patients of a family with D178N-129M/M	All FFI
Chen et al.,2018^[13]^	Chinese	7 patients with D178N-129M	4 CJD phenotype, 3 FFI phenotype

As summarized above, there are a variety of phenotypes in patients with this haplotype, the pathology change in these patients is crucial to reveal a possible explanation behind this dissociated phenomenon. Hence, there is a point of view suggesting that because D178N-129M patients manifest a certain type of prion diseases, which comprise a clinical and pathological overlap between FFI and CJD, so probable representing a spectrum disease group rather than two discrete diseases [12,16]. Still, there is a hypothesis based on some autopsies of D178N-129M patients demonstrating that among these clinical diagnosed CJD, pathology changes prone to be FFI indeed [17]. However, this point of view may not explain all D178N-129M patients mimic fCJD but truly are FFI, though it makes a continuous spectrum of FFI and CJD instead of two separate entities more convincible.

In a molecular level, it is proved that PrP with D178N mutation was more susceptible to oxidation and this process can enhance aggregation and neurotoxicity of mutant PrP [18], whereas the molecular mechanism of different D178N-129M/V haplotypes with different phenotypes is still not clear. It leaves great space to explore for the mechanisms back of this genotype-phenotype pattern.

## Data Availability

The datasets supporting the conclusions of this article are included in the article.

## Declarations

**Ethics approval and consent to participate**:

The study was approved by Ruijin hospital, Shanghai Jiao Tong University School of Medicine and the patient gave written informed consent prior to obtain the data.
